# Relationship Between Neutrophil/Albumin Ratio and Early Mortality After Major Lower Extremity Amputation

**DOI:** 10.7759/cureus.17733

**Published:** 2021-09-05

**Authors:** Ali Eray Günay, Mehmet Ekici

**Affiliations:** 1 Orthopaedics and Traumatology, Kayseri City Education and Research Hospital, Kayseri, TUR

**Keywords:** nar, limb amputation, lea, diabetic foot infection, neutrophil, albumin

## Abstract

Introduction

Diabetic foot infection is a condition that affects the patient's life, may cause limb loss, and has a high mortality. Too many parameters were used for predicting early mortality but the gold standard method wasn't described. Neutrophil lymphocyte ratio (NLR) is universally accepted as a predictive value for amputation-free survival and mortality. NLR increases due to inflammation-induced neutrophilia and lymphopenia related to cortisol-induced stress. Increasing in the neutrophil albumin ratio is expected due to decreasing albumin levels because albumin is a negative acute-phase reactant. The aim of this study is to investigate the sensitivity and value of the neutrophil albumin ratio (NAR) for early mortality after major lower extremity amputation (LEA).

Methods

Following the approval of the ethics committee, 87 patients who underwent major LEA between May 2018 and May 2020 were analyzed for the study. White blood cell (WBC), neutrophil, lymphocyte, C-reactive protein (CRP), creatinine, albumin, platelet, and hemoglobin values on the day prior to surgery were recorded. NLR was calculated as the ratio of neutrophil count to lymphocyte count, NAR as the ratio of neutrophil count to albumin value, CRP/albumin ratio (CAR) as the ratio of CRP value to albumin value, and platelet lymphocyte ratio (PLR) as the ratio of platelet count to lymphocyte count. Each parameter was also recorded in the postoperative second week.

Results

Of the patients included in the study, 52 were men (59.8%) and 35 were women (40.2%). It was determined that 29 of 87 patients (33.3%) died within the first year. The relationship between post-operative NAR value and early mortality is examined. The area under the curve was calculated as 0.873. When the cut-off value was applied as 0.265, the sensitivity was found as 88% and specificity as 76%.

Conclusions

Higher neutrophil/albumin ratio after lower extremity amputation was associated with early mortality after extremity amputation. This parameter can help predict mortality. The cut-off value was determined as 0.265, the sensitivity was found as 88%, and specificity as 76%.

## Introduction

Amputations performed at the proximal ankle level are called major lower extremity amputation (LEA) [1]. Major LEA is a surgical procedure with high morbidity and mortality rates. The incidence of nontraumatic major lower extremity amputation is 12-50 per 100000 annually [2]. After non-traumatic major amputation, the five-year mortality rate reaches 40% [3].

Patients requiring major amputation on the basis of atherosclerosis and diabetes mellitus are generally elderly and have multiple comorbidities [4]. Critical limb ischemia is a clinical condition characterized by nonhealing ulcers especially in the lower extremities, gangrene, and pain in tissues other than the ischemic area [2]. Severe inflammation occurs in case of ischemic tissue damage. In this case, the neutrophil lymphocyte ratio (NLR) increases due to inflammation-induced neutrophilia and lymphopenia related to cortisol-induced stress [5-7]. Similar to the NLR, the platelet/lymphocyte ratio is also one of the inflammatory response values that increase with systemic inflammation. They have previously been used as a prognostic biomarker in various diseases [4,8]. Limb ischemia is an atherosclerotic disease associated with an inflammatory response. In previous studies, C-reactive protein (CRP), platelet aggregation, and NLR were defined as effective predictive values for limb ischemia [9,10]. In addition, NLR is universally accepted as a predictive value for amputation-free survival and mortality [11].

Serum albumin level is a negative acute-phase reactant. Its level in the blood decreases with systemic inflammatory response [12]. Although the neutrophil/albumin ratio (NAR) has been used as a prognostic factor in different areas, there is no study on the predictive effect of NAR on limb ischemia and related amputations [13,14].

The hypothesis of this study is that increased systemic response is associated with early mortality following major LEA. The rising level of NAR may predict mortality risk. The aim of this study is to investigate the sensitivity and value of the NAR for early mortality after major LEA.

## Materials and methods

Following the approval of the local ethics committee, 163 patients who underwent major LEA at our institution between May 2018 and May 2020 were retrospectively analyzed for the study.

Patients who did not benefit from conservative treatment and other surgical procedures (i.e., revascularization) and had undergone major amputation due to diabetic foot ulcer or peripheral artery disease (PAD) were included in the study. Patients who underwent amputation secondary to trauma, underwent bilateral amputation, had sepsis, had a history of malignancy, used corticosteroids, and patients whose blood white blood cell (WBC) count was less than 4x109/L or more than 25x109/L before surgery were excluded from the study. A total of 76 patients were excluded from the study due to exclusion criteria.

As surgical procedures, below-knee amputation, above-knee amputation, or knee disarticulation were performed on the patients. Demographic data, such as age, gender, follow-up time, postoperative survival was evaluated.

Patients were divided into two groups according to the cut-off value of NAR level found by receiver operating characteristic (ROC) analysis after surgery. Those with NAR levels below the cut-off value were named group one, and those with a higher cut-off value were named group two.

WBC, neutrophils, lymphocyte count, CRP, creatinine, albumin, platelets (PLT), and hemoglobin (HGB) values from the venous blood sample drawn on the day prior to surgery were retracted from the records. Venous blood sample results drawn in the second week after surgery were determined as a control value. NLR was calculated as the ratio of neutrophil count to lymphocyte count, NAR as the ratio of neutrophil count to albumin value, CRP/albumin ratio (CAR) as the ratio of CRP value to albumin value, and PLR as the ratio of platelet count to lymphocyte count. Each parameter was recorded separately as pre-op and post-op second week.

The data was transferred to the computer environment and analyzed with IBM Corp. Released 2013. IBM SPSS Statistics for Windows, Version 22.0. Armonk, NY: IBM Corp. The compliance of quantitative data to normal distribution was tested with the Shapiro-Wilk test. Pearson’s Chi-Square was used for the analysis of categorical data, and Mann-Whitney U and Kruskal-Wallis tests were used for the analysis of quantitative data that did not show normal distribution. ROC analysis was used to calculate the predictive value of pre-operative and post-operative NAR, PLR, CAR, and NLR specificity and sensitivity ratios. Results with a p-value less than 0.05 were considered statistically significant. 

## Results

Of the 87 patients included in the study, 52 were men (59.8%) and 35 were women (40.2%). The mean age of the patients was found to be 70.63 ± 1.27 (44-92) years. 23 patients (26.4%) were amputated due to PAD and 64 patients (73.6%) were amputated due to diabetic foot ulcers. The mean hospital stay was 21.95 ± 1.79 days.

It was determined that 19 patients (21.8%) died in the first month after surgery, nine died in the first to sixth months, and one patient died in the 12th month, 29 of 87 patients (33.3%) died within the first year. It was determined that two of the patients who died in the first month died within the first 24 hours after the operation, 16 died in the hospital before discharge, and three died at home after discharge. The mean follow-up period of the patients who did not die was found to be 15.6 ± 1.13 months.

As a surgical procedure, 73 patients (83.9%) underwent below-knee amputation, 12 patients (13.8%) underwent above-knee amputation, and two (2.3%) underwent knee disarticulation. The mean operation time was 68.28 ± 2.59 minutes.

Pre-op and post-op blood parameters of the patients are shown in Table 1. Pre-op and post-op NAR, CAR, NLR, and PLR cut-off values of the patients were calculated for early mortality. Post-op mortality numbers for each value, including the patients below and above the cut-off value, are given in the table. Since two patients died within 24 hours postoperatively, albumin values were not found in the records (Table 2).

**Table 1:** Comparision of the pre-operative and post-operative blood parameters NLR: Neutrophil/Lymphocyte ratio, NAR: Neutrophil /Albumin ratio, CAR: CRP/Albumin ratio, PLR: Platelet/ Lymphocyte ratio, g: gram, dL: deciliter, mg: milligram, L: Liter, WBC: White Blood Cell

		Preop			Postop		p
	Median	Min	Max	Median	Min	Max	
WBC (x10^9^/L)	12.36	6.53	24.10	8.48	4.04	15.29	<0.001
Neutrophil (x10^9^/L)	9.58	3.96	22.33	5.95	2.05	13.15	<0.001
Lymphocyte(x10^9^/L)	1.51	0.57	3.14	1.56	0.54	2.86	0.930
Hemoglobin (g/dL)	10.20	7.1	13.4	9.8	7.4	13.4	0.041
Platelet (10^3^/mm^3^)	320	73	631	325	147	846	<0.001
CRP (mg/L)	136.1	20.20	379.4	51.5	7.8	211.2	<0.001
Albumin (g/dL)	25.4	14	36.4	27	22.7	37	0.390
Creatinine (mg/L)	0.95	0.45	5.36	0.88	0.37	6.07	0.045
NLR	6.04	1.62	19.93	3.39	1.21	24.35	<0.001
PLR	207.79	100	555.91	218.18	115.54	409.41	0.876
NAR	0.34	0.10	1.08	0.20	0.07	0.52	<0.001
CAR	5.24	0.65	20.50	1.96	0.26	8.1	<0.001

**Table 2:** ROC analysis results and cut-off values of preoperative and postoperative NAR, CAR, PLR, NLR, and post-operative mortality rates AUC: Area under curve, NLR: Neutrophil/Lymphocyte ratio, NAR: Neutrophil/Albumin ratio, CAR: CRP/Albumin ratio, PLR: Platelet/ Lymphocyte ratio

					Mortality		Total	p
	AUC	Cut-off	Sensitivity	Spesivity	No n (%)	Yes n (%)		
NAR								
Pre-operative	0.707	0.365≥	62	63	36 (%62.0)	11 (%38.9)	47	0.041
		0.365<			22 (%38.0)	18 (%61.1)	40	
Post-operative	0.865	0.265≥	88	76	43 (%74.1)	3 (%11.1)	46	<0.001
		0.265<			15(%25.9)	24(%88.9)	39	
NLR								
Pre-operative	0.746	6.37≥	93	52	30 (%51.7)	2 (%6.9)	32	0.041
		6.37<			28 (%48.3)	27 (%93.1)	55	
Post-operative	0.883	7.06≥	81	85	49 (%84.5)	5 (%18.5)	54	<0.001
		7.06<			9 (%15.5)	22 (%71.5)	31	
PLR								
Pre-operative	0.547	247.28≥	51	64	37 (%63.8)	13 (%44.8)	50	0.176
		247.28<			21 (%36.2)	16 (%55.2)	37	
Post-operative	0.611	191.71≥	74	44	25 (%43.1)	7 (%25.9)	32	0.154
		191.71<			33 (%56.9)	20 (%74.1)	53	
CAR								
Pre-operative	0.540	2.79≥	82	23	13 (%24.1)	6 (%22.2)	19	0.778
		2.79<			41 (%75.9)	21 (%77.8)	62	
Post-operative	0.790	3.07≥	78	71	34 (%69.4)	6 (%25.0)	40	<0.001
		3.07<			15 (%30.6)	18 (%75.0)	33	

**Figure 1:**
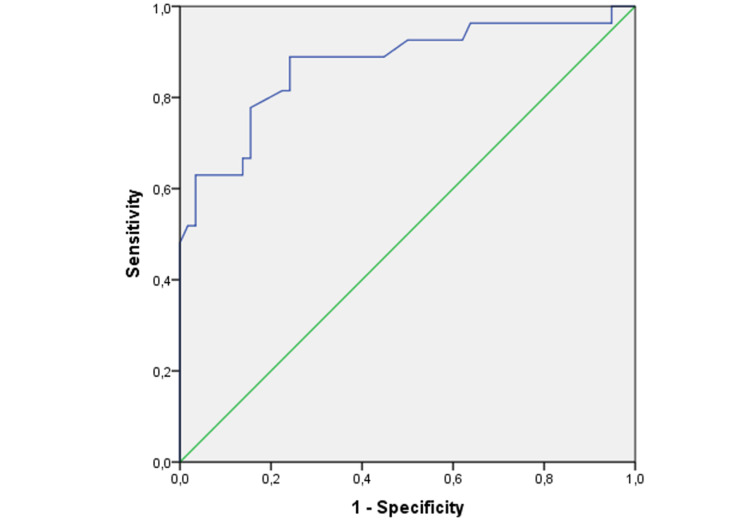
ROC analysis graphic for post-op NAR NAR: Neutrophil/Albumin ratio ROC: Receiver operating characteristic

The relationship between post-op NAR value and early mortality is shown in Figure 1.

The area under the curve was calculated as 0.873. When the cut-off value was applied as 0.265, the sensitivity was found as 88% and specificity as 76%. For the NLR pre-op cut-off value, sensitivity was calculated as 93% and specificity as 52% (Table 2). According to logistic regression analysis results, it was observed that a postoperative NAR value over 0.265 increases the risk of death 11.5 times (Table 3). Distributions of demographic and laboratory results after grouping with post-operative NAR cut-off values are shown in Table 4 and Table 5. 

**Table 3:** Effect of postoperative NAR value on mortality according to logistic regression analysis NAR: Neutrophil/Albumin Ratio, CAR: C-reactive protein/Albumin Ratio, OR: Odds Ratio, CI: Confidence Interval

	Cut Off	n	Mortality (%)	OR (CI 95%)
NAR postoperative	0.265≥	46	3 (6.5%)	1.0
	0.265<	39	24 (61.5%)	11.5 (2.6 – 50.3)
CAR postoperative	3.070≥	40	6 (15.0%)	1.0
	3.070<	33	18 (54.5%)	2.3 (0.6 – 8.9)

**Table 4:** Distributions of demographic and laboratory results after grouping with post-operative NAR cut off value NLR: Neutrophil/Lymphocyte ratio, NAR: Neutrophil/Albumin ratio, CAR: CRP/Albumin ratio, PLR: Platelet/Lymphocyte ratio, g: gram, dL: deciliter, mg: milligram, L: Liter, WBC: White Blood Cell, CRP: C-reactive protein

	Group 1 (n=46)			Group 2 (n=39)			
	Median	Min	Mix	Median	Min	Max	p
Age (years)	67.50	44	91	77	49	92	0.050
Surgery Duration (min)	72	25	140	64	30	170	0.148
Follow Up Time (months)	15.50	1	31	15.5	1	31	<0.001
Hospitalization (day)	20.50	4	78	21	6	53	0.251
WBC Pre-op (x10^9^/L)	11.67	6.53	24.10	12.90	6.61	19.36	0.027
WBC Post-op (x10^9^/L)	7.71	4.04	11.40	10.61	8.50	14.95	<0.001
Neutrophil Pre-op (x10^9^/L)	8.71	3.96	22.33	10.09	1.67	17.24	0.022
Neutrophil Post-op (x10^9^/L)	4.88	2.05	8.55	7.97	5.40	12.44	<0.001
Lymphocyte Pre-op(x10^9^/L)	1.48	0.32	3.14	1.50	0.42	3.40	0.195
Lymphocyte Post-op(x10^9^/L)	1.63	0.49	2.86	1.56	0.73	2.60	0.006
NAR Pre-op	0.34	0.10	1.08	0.37	0.07	1.01	0.060
NAR Post-op	0.17	0.07	0.25	0.32	0.26	0.41	<0.001
Hb Pre-op (g/L)	10.60	7.10	14.40	9.70	9.10	13.40	0.774
Hb Post-op (g/L)	9.80	7.40	13.40	9.70	8.20	13.30	0.478
PLT Pre-op (10^3^/mm^3^)	319	66	664	272	145	570	0.815
PLT Post-op (10^3^/mm^3^)	297	53	846	312	100	629	0.161
Creatinine Pre-op (mg/L)	0.92	0.44	8.02	1.18	0.63	5.36	0.031
Creatinine Post-op (mg/L)	0.82	0.28	7.45	1.00	0.66	6.07	0.013
CRP Pre-op (mg/L)	121.10	16.50	393.50	162.00	88.80	322.00	0.167
CRP Post-op (mg/L)	49.20	7.00	175.40	88.15	40.40	211.20	<0.001
Albumin Pre-op (g/dL)	25.00	16.00	36.20	25.00	17.00	33.10	0.965
Albumin Post-op (g/dL)	26.90	19.00	37.00	24.50	16.00	31.00	<0.001

**Table 5:** Distribution of gender, diabetes mellitus prevalence, amputation level, and mortality day

	Group 1	Group 2	p
Gender			
Male	26	25	0.512
Female	20	14	
Diabetes Mellitus	36/46	27/39	0.457
Amputation			
Below Knee	41	30	0.152
Above and Knee	6	9	
Mortality			
30 Days>	1	16	0.535
30 Days≤	2	8	

## Discussion

Mortality after major LEA is a serious issue. It is important to be able to predict this situation in order to prevent medicolegal problems and to manage treatment. Mortality in the first 30 days after major LEA was reported between 7%-30%, and one-year mortality was reported between 19% and 48% [15-20]. In our study, the first-month mortality after surgery was 21.8%, and the one-year mortality was 33.3%.

NLR is a known proinflammatory parameter. Luo et al. showed that the NLR value on the seventh day after treatment in critical limb ischemia is an independent predictive value for the risk of amputation [21]. Teperman et al. [8] showed that there is a significant relationship between NLR and the severity of lower extremity PAD. Fest et al. [22] showed that the NLR value is an underlying disease-independent risk factor for mortality in the whole population. In our study, the cut-off value of NLR for early mortality was 6.37 (93% sensitivity, 52% specificity) for preoperative values and 7.06 (81% sensitivity, 85% specificity) for postoperative values. These values demonstrate that the postoperative NLR value is a strong predictor of early mortality.

In this study, the ratio of postoperative NAR value was found to have an independent predictive value for early mortality. NAR postop cut-off value for early mortality was found to be 0.265 (88% sensitivity, 76% specificity). The sensitivity and specificity of the preop NAR value were found to be lower than the postop NAR value. Earlier NAR has been used in few studies. Hwang et al. [23] showed that patients with severe sepsis with high delta neutrophil/serum albumin ratio (DNI/A) had higher 28-day early mortality rates, and Peng et al. [24] showed the relationship between NAR value and mortality after cardiogenic shock.

Decrease in neutrophil value and increase in albumin level result in a decrease in NAR value. It was observed that the neutrophil value was lower and the albumin value was higher in patients with low NAR values after surgery, and these differences were considered statistically significant. Serum albumin level is a nutritional marker and a negative acute phase reactant for inflammation [11, 25]. Yeşil et al. found that in patients who were followed up for diabetic foot ulcers, serum albumin levels of patients who underwent amputation were lower than patients who recovered [26].

Thrombocytosis and lymphopenia correlate with the severity of systemic infection, and PLR is a new infection marker that includes both hematological values. Taşoğlu et al. reported that the extremity survival time was significantly reduced in patients with a PLR value >160 in PAD-induced extremity ischemia [27]. Yaprak et al. concluded that PLR is a superior marker to NLR in determining early mortality in patients with end-stage renal disease [28]. In our study, the sensitivity of preoperative PLR value for early mortality was 51% and its specificity was 64%. The sensitivity of postop PLR was 74% and its specificity was 44%.

CRP is an acute phase reactant. It has been shown that there is a potential relationship between rising CRP value and major amputation [23,29]. CAR in critically ill patients and malignancies is a prognostic factor based on systemic infection [29]. Süleymanoğlu et al. [30] showed the CAR value as an independent predictor in the prognosis of PAD. In our study, the cut-off value for preop CAR was 2.79 (sensitivity 82%, specificity 23%), and the postop CAR cut-off value was 3.07 (sensitivity 78%, specificity 71%). NLR value is a superior marker for mortality after major amputation compared to CAR and PLR.

The limitations of the study were that it was conducted on a relatively small group and was a single center retrospective study.

## Conclusions

In conclusion, a higher neutrophil/albumin ratio after lower extremity amputation was associated with early mortality after extremity amputation. When the cut-off value for NAR was accepted as 0.265, the sensitivity was found to be 88% and the specificity as 76%. Prospective studies with larger case series aiming to reveal which of the factors that increase this rate are more effective on mortality are needed.
